# Vaccination Recommendation Patterns and Associated Factors Among Children with Special Health Care Needs: A Cross-Sectional Study in District-Level Immunization Services in China

**DOI:** 10.3390/vaccines13111145

**Published:** 2025-11-07

**Authors:** Chenglian Wang, Li Zhang, Binyue Xu, Xiaolan Fu, Li Fu, Bowen Li, Wang Ju, Qingyu Li, Sitong Luo

**Affiliations:** 1 Changshou Center for Disease Control and Prevention, Chongqing 401220, China; 2 Chongqing Municipal Center for Disease Control and Prevention, Chongqing 400016, China; 3 Changshou Traditional Chinese Medicine Hospital, Chongqing 401220, China; 4 Vanke School of Public Health, Tsinghua University, Beijing 100084, China

**Keywords:** children with special health care needs, vaccination recommendation, immunization consultation, primary health care, vaccine deferral, China

## Abstract

**Background**: Children with special health care needs (CSHCN) are at heightened risk of delayed or missed vaccinations because of clinical complexity and provider uncertainty. Although China has issued expert consensus guidelines and established immunization advisory services, little is known about vaccination decision-making within routine district-level immunization services. This study examined vaccination recommendation patterns and factors associated with deferral or non-recommendation among CSHCN in these settings. **Methods**: This cross-sectional study was conducted between 1 April 2023, and 31 March 2024, in Changshou District, Chongqing, China, encompassing 18 primary health centers, five general hospitals and one special hospital. Children aged 0–18 years identified by physicians as having conditions potentially affecting vaccination decisions and with at least one documented vaccination-related consultation were included. Clinical characteristics and physician recommendations (“recommended,” “temporarily deferred,” “not recommended”) were recorded via the national Epidemiological Dynamic Data Collection (EDDC) system; subsequent vaccination uptake was verified using the Chongqing Immunization Information Management System. Logistic regression identified factors associated with deferral or non-recommendation. **Results**: A total of 761 participants were included in the analysis, with a median age of 12 months (IQR: 1–47). Among all vaccine-specific recommendations, 55.2% were classified as “recommended,” 43.4% as “temporarily deferred,” and 1.5% as “not recommended”. Deferral proportions varied markedly, highest in respiratory (73.6%) and immunologic (42.1%) conditions and lowest in neonatal disorders (4.0%). Compared with 0–6-month-olds, children aged 7–12 months (adjusted odds ratio [aOR] = 5.26, 95% CI 2.30–12.33) and >13 months (aOR = 13.48, 95% CI 6.69–28.34) were more likely to receive deferral or non-recommendation; multimorbidity also increased odds (aOR = 20.68, 95% CI 11.26–40.10). Consultations at primary health centers were associated with a lower likelihood of deferral or non-recommendation (aOR = 0.26, 95% CI 0.15–0.45). **Conclusion**: Vaccination recommendations for CSHCN vary considerably across clinical profiles and facility types in routine immunization settings. Despite national initiatives, many vaccine doses remain deferred or not recommended. Disease-specific guidelines, enhanced provider training, and context-adapted decision-support tools are needed to promote timely and equitable immunization for this medically vulnerable population.

## 1. Introduction

Vaccination has been one of the most cost-effective public health interventions to prevent childhood mortality and morbidity from infectious diseases [1]. Since the initiation of the planned immunization program in 1978, China has established a nationwide network of child vaccination clinics reaching the village level, with vaccinators primarily being doctors or nurses. The country now operates a dual-tier vaccination system: vaccines included in the National Immunization Program (NIP) are provided free of charge and are mandatory for all children according to national guidelines, while non-NIP vaccines require out-of-pocket payment. The NIP currently includes 14 vaccines targeting 15 infectious diseases, and the national coverage rate among children under six years of age has consistently exceeded 99%, contributing substantially to the protection of child health [2].

Despite these achievements in immunization coverage, certain vulnerable groups remain underserved. Children with special health care needs (CSHCN) refers to a population of children who either have or are at an elevated risk for chronic physical, developmental, behavioral, or emotional conditions [3]. Weakened immunity and repeated hospitalizations further increase their susceptibility to vaccine-preventable diseases. Studies from the United States and Italy have reported that CSHCN were more likely to have lower vaccination coverage and experience higher proportion of delayed or missed vaccinations compared to the general pediatric population [4,5,6,7]. These disparities may result from provider hesitancy, parental concerns, or a lack of clear immunization guidance for this population.

In China’s immunization practice, CSHCN are operationally defined as those with temporary or chronic physiological (e.g., prematurity, metabolic abnormalities) or pathological conditions (e.g., chronic organ disease, immune dysfunction) requiring individualized vaccination assessment [8]. Each year, approximately 2.12 million newborns are affected by birth defects or immune-related conditions in China [8]. Existing studies also shown that CSHCN generally have lower vaccination coverage than their healthy peers, with frequent delays or missed doses. In Zhejiang Province, only 75.0% of CSHCN were recommended full vaccination, while 21.2% were advised to defer specific vaccines and 3.8% to delay all vaccinations [9,10]. Similar patterns were observed in Suzhou City, where 66.9% received full vaccination recommendations, 29.0% partial, and 4.1% were advised to delay all vaccines [11]. Furthermore, older age, a history of adverse events following immunization (AEFI), and multiple comorbidities were significantly associated with higher odds of receiving deferral recommendations.

Both internationally and in China, efforts have been made to guide immunization practices for CSHCN. Guidelines and expert consensus documents have been developed to support clinical decision-making in this population [8,12,13,14,15]. Additionally, several Chinese cities have established immunization advisory clinics and referral systems to assist with the evaluation and vaccination of CSHCN. Nevertheless, many vaccination providers—especially those working in district-level immunization services—lack the professional training or clinical authority to evaluate vaccination eligibility for this population, leading to inconsistent and sometimes delayed immunization recommendations.

Existing research in China has mainly focused on specialized immunization advisory clinics in tertiary or urban centers, with limited evidence on vaccination practices for CSHCN in routine district-level immunization services. District-level immunization services constitute the primary point of vaccination delivery for most children in China and are responsible for implementing national immunization guidelines in real-world settings. Focusing on this level allows assessment of vaccination recommendations, as they occur under routine resource and training conditions. Compared with previous studies conducted in tertiary or urban advisory settings (e.g., Zhejiang and Suzhou), the present study examines vaccination recommendation patterns in routine district-level clinics, where provider training, health system capacity, and access to specialist consultation are often more constrained and heterogeneous. This contextual focus reflects the settings in which most children in China actually receive vaccination care.

This study aimed to examine vaccination recommendation patterns and identify factors associated with deferral or delay among CSHCN attending district-level immunization services in China, thereby providing evidence from routine immunization settings. The findings are expected to provide empirical insights into physician decision-making and system-level disparities, informing strategies to improve equitable vaccination for CSHCN.

## 2. Method

### 2.1. Study Design and Participants

This study employed a cross-sectional design, and vaccination outcomes were retrospectively verified via record linkage between the Epidemiological Dynamic Data Collection (EDDC) system and the Chongqing Immunization Information Management System. It was conducted from 1 April 2023 to 31 March 2024 in Changshou District, Chongqing, China. Changshou is a mid-sized administrative district in western China that combines urban and rural communities and operates a well-established district-level immunization network. The district maintains an independent electronic immunization information management system linked to the national platform, allowing accurate record linkage between physician recommendations and vaccination outcomes.

All 18 primary health centers, 5 general hospitals, and 1 specialized hospital in the district participated, therefore representing all routine immunization facilities in the area. Eligible participants were children aged 0–18 years who visited immunization clinics during the study period and were identified by the attending physician as having at least one special health care need. The study was population-based rather than sample-based, consistent with full facility coverage, and the number of cases in the area during the study period determined the sample size [16]. This approach ensured adequate statistical precision and minimized sampling variability.

Children were included if their immunization records met all of the following criteria: (1) documentation of at least one vaccination-related clinical consultation during the study period; (2) a vaccination recommendation provided by a licensed physician (including recommendation to vaccinate, temporary deferral, or non-recommendation); and (3) a clearly recorded diagnosis or disease classification. Children were excluded if they lacked documentation of a special health care need or vaccination advice, or if essential information such as sex, date of birth, disease type, or consultation outcome was missing. Duplicate entries (defined as repeat consultations for the same child identified by unique administrative codes) were also excluded.

**Figure 1:**
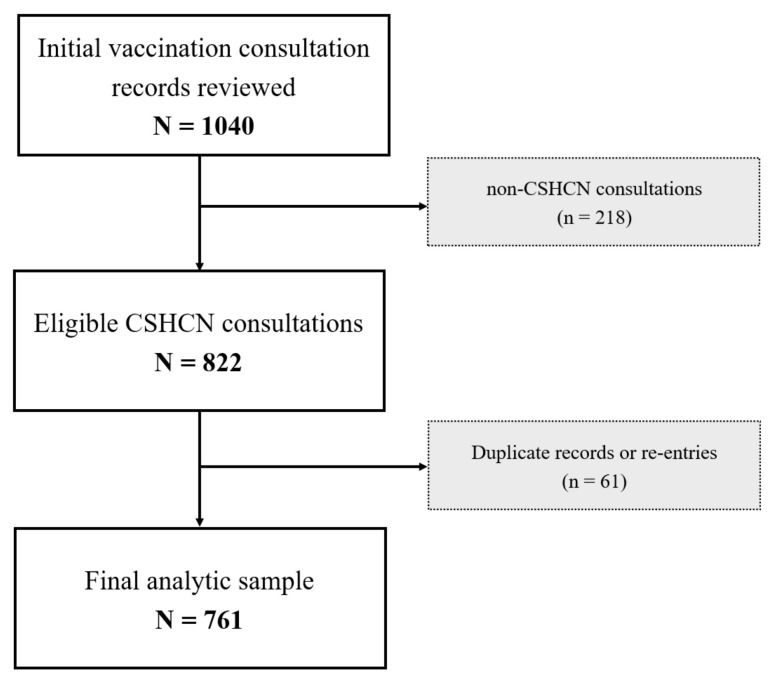
Flowchart of this study.

A total of 1040 vaccination consultation records were reviewed. After excluding 218 consultations not meeting the CSHCN definition and 61 duplicate records, the final analytic sample included 761 eligible participants as shown in Figure 1.

### 2.2. Procedures

At each participating health facility, when a child presented for vaccination, the attending physician first assessed whether the child had a special health care need that could potentially affect vaccination decisions. For children identified as having such conditions, vaccination personnel completed the Special Health Care Need Child Information Registration Form using the Epidemiological Dynamic Data Collection (EDDC) platform-an electronic data entry system developed and maintained by the Chinese Center for Disease Control and Prevention (China CDC). The EDDC platform includes built-in data fields and logical checks to improve completeness and consistency.

The registration form collected both sociodemographic and clinical information. Sociodemographic variables included the child’s sex, date of birth, and an administrative registration code that indicated the level and type of healthcare facility. Clinical data were recorded by attending physicians using a standardized electronic form embedded in the EDDC system (Supplementary File S1). The form contained a predefined list of special health conditions grouped into major disease categories (e.g., neonatal disorders, congenital heart disease, respiratory diseases, and others). Within each category, physicians selected one or more specific diagnoses from the list (e.g., pathological neonatal jaundice, patent ductus arteriosus, eczema, and others) and recorded the disease stage at the time of consultation (acute, stable, recovered, or other) as well as the date of first diagnosis. Free-text fields were available only for “other” conditions not listed. Other information includes presence of comorbidities (yes/no), and the date of registration.

For each relevant vaccine and dose, physicians recorded a vaccination recommendation based on the child’s health condition and national immunization technical guidelines. Recommendations were categorized as “recommended,” “temporarily deferred,” or “not recommended.” In this study, “temporarily deferred” referred to postponement of a scheduled vaccine dose pending re-evaluation at a later visit, whereas “not recommended” indicated that the vaccine was contraindicated or considered inappropriate for the child. No unified training protocol was implemented across sites, and vaccination recommendations were made according to routine clinical practice within each facility.

To evaluate subsequent vaccination behavior, follow-up data were extracted from the Chongqing Immunization Information Management System at the end of the study period. This allowed verification of whether each vaccine had ultimately been administered and the timing of vaccination. Timeliness was assessed according to the national immunization schedule, with delayed vaccination defined as administration beyond the recommended age window for each dose.

### 2.3. Ethics Approval and Informed Consent

This study was reviewed and approved by the Ethics Committee of the Chongqing Municipal Center for Disease Control and Prevention (No: CQCDCLS (2021)006). Written informed consent was obtained from the legal guardians of all participating children at the time of vaccination consultation.

### 2.4. Statistical Analysis

Descriptive analyses were first conducted to summarize sample characteristics, including age, sex, and level of health facility. For each disease category (e.g., congenital, respiratory, neurological), the three most common specific conditions were identified and reported; less frequent diagnoses within each category were grouped into an “other” category. Vaccination recommendations were further described by disease category. For vaccines and doses with clinical advice of deferral or non-recommendation, we calculated the proportion of children who eventually received the corresponding vaccine and, where applicable, assessed the timeliness of vaccination.

To identify factors associated with clinical vaccination recommendations, we dichotomized the original outcome into a binary variable: “recommended” versus “temporarily deferred or not recommended,” consistent with our conceptual aim of capturing physicians’ real-world decision-making thresholds for proceeding with vaccination among CSHCN. This grouping allowed comparison between children fully cleared for vaccination and those with any form of restriction. Univariate logistic regression models were first fitted for each independent variable, and odds ratios (ORs) with 95% confidence intervals (CIs) were reported. All variables—including demographic factors (age, sex, facility level), disease category, comorbidity status, and history of adverse events following immunization (AEFI)—were then entered into multivariable logistic regression models to assess independent associations, and adjusted odds ratios (aORs) with corresponding 95% CIs were calculated. Two-sided *p* values < 0.05 were considered statistically significant. All statistical analyses were performed using R software (version 4.4.1).

## 3. Result

### 3.1. Sample Characteristics

Among 761 participants in the study, the median age of the study participants was 12 months (IQR = 1–47), with the largest proportion aged 0–6 months (n = 296, 38.8%). Slightly more than half of them were assigned male sex at birth (n = 411, 54.0%). One-third of the children were in the acute phase of their illness (n = 258, 33.9%). Nearly all had no multimorbidity (n = 741, 97.4%) and most received care in general hospitals (n = 560, 73.6%). In most cases (n = 527, 69.3%), the vaccination consultation involved NIP vaccines. Detailed characteristics are presented in Table 1.

### 3.2. Distribution of Diseases

Among the Children with special health care needs, 9 disease types and 122 specific diseases were reported. The most commonly reported were respiratory diseases (n = 317, 41.7%), followed by neonatal disorders (n = 190, 25.0%), immunological disorders (n = 105, 13.8%), and infectious diseases (n = 62, 8.1%). Fewer cases were reported under hematologic and neoplastic disorders (n = 23, 3.0%), neuromuscular disorders (n = 19, 2.5%), chronic conditions (n = 17, 2.2%), and congenital heart disease (n = 16, 2.1%), and other special health conditions (n = 12, 1.6%).

Among specific diagnoses, acute upper respiratory infection was the most frequently reported condition, affecting 207 children (27.2%). Pathological neonatal jaundice was documented in 97 children (12.7%), and prematurity in 62 (8.1%). Atopic dermatitis was identified in 52 cases (6.8%), and acute gastroenteritis in 17 (2.2%). The detailed distribution of conditions is presented in Table 2.

### 3.3. Vaccination Recommendations by Disease Type

**Figure 2:**
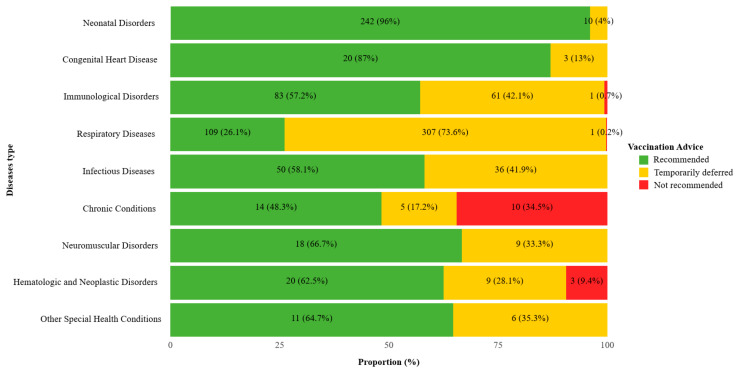
Vaccination recommendations distribution by disease type among Children with special health care needs in the study. Distribution of vaccination recommendations among children with special health care needs by disease type. Statistical significance of differences in recommendation distributions across disease categories was assessed using the *χ^2^* test (*p* < 0.001).

Across all vaccine recommendations among CSHCN, 55.2% (567/1028) were recommended for vaccination, 43.4% (446/1028) were temporarily deferred, and 1.5% (15/1028) were not recommended. The distribution of vaccination advice differed significantly across disease categories (*χ^2^* = 577.5, *p* < 0.001). In the respiratory disease group, 307 out of 417 doses (73.6%) were temporarily deferred. Among children with immunological disorders, 42.1% of doses (61/145) were deferred, and 0.7% (1/145) were not recommended. Notably, children with chronic conditions showed the highest proportion of non-recommendation, with 10 out of 29 doses (34.5%) classified as not recommended. Children with neonatal disorders and congenital heart disease had the lowest proportion of deferred or non-recommended doses—10/252 (4.0%) and 3/23 (13.0%) doses, respectively, were deferred or not recommended (Figure 2).

### 3.4. Vaccination Recommendation Patterns Across Doses

Table 3 presents the distribution of vaccination recommendations across different dose levels for each vaccine included in China’s National Immunization Program (NIP). For HBV, IPV, OPV, and DTaP, the proportion of doses being recommended, temporarily deferred, or not recommended varied significantly by dose number. For the HBV vaccine, 95.9% of children were recommended to receive the first dose (71 out of 74) and the second dose (141 out of 147). For the third dose, the recommendation proportion declined to 45.5% (10 out of 22), while 50.0% were temporarily deferred and 4.5% were not recommended. For the fourth dose, none were recommended; 80.0% (4 out of 5) were temporarily deferred, and 20.0% were not recommended. Results for non-NIP vaccines are provided in Table S1. This pattern was not observed for the other vaccines.

### 3.5. Vaccination Coverage of NIP Vaccines with Temporarily Deferred or Not Recommended Status

A total of 313 vaccination opportunities involving NIP vaccines with clinical advice of temporary deferral or non-recommendation were recorded (Table 4). Of these, 210 (67.1%) resulted in actual vaccination, while 103 (32.9%) did not. The follow-up period for assessing vaccination uptake lasted until the end of the study. Among those who were eventually vaccinated, varying degrees of delay were observed relative to the date of clinical advice. Non-vaccination counts ranged from 0 (0.0%) to 33 (87.5%).

### 3.6. Factors Associated with Vaccination Recommendations

Multivariable logistic regression analysis identified that, compared to children aged 0–6 months, those aged 7–12 months had higher odds of being temporarily deferred or not recommended (adjusted odds ratio [aOR] = 5.26, 95% CI: 2.30–12.33), and the association was even stronger for those older than 13 months (aOR = 13.48, 95% CI: 6.69–28.34). Children with multimorbidity were also more likely to receive a deferred or non-recommendation (aOR = 20.68, 95% CI: 11.26–40.10). Compared with those diagnosed with neonatal disorders, children with respiratory diseases had significantly higher odds of being deferred or not recommended (aOR = 4.25, 95% CI: 1.66–11.69), followed by those with infectious diseases (aOR = 3.02, 95% CI: 1.01–9.63). No significant associations were observed for other disease types after adjustment. Children who received vaccination services at primary health centers were less likely to be deferred or not recommended than those at general hospitals (aOR = 0.26, 95% CI: 0.15–0.45). Other variables were not significantly associated with recommendation in the adjusted model (Table 5).

## 4. Discussion

This study provides a comprehensive assessment of vaccination recommendation patterns for children with special health care needs (CSHCN) within routine district-level immunization services in China. We analyzed 1028 vaccine–dose level recommendations across 761 children and found that only 55.2% of doses were recommended by clinicians, whereas 43.4% were temporarily deferred and 1.5% were not recommended. These proportions indicate that restrictions to vaccination in routine practice are driven predominantly by precautionary deferral rather than by permanent contraindication. Additionally, these proportions are lower than those reported in previous studies from Zhejiang Province and Suzhou City [10,11], indicating that immunization challenges may be more pronounced in under-resourced district-level immunization services. Our findings highlight the importance of strengthening capacity and providing context-specific clinical decision support to ensure that children with complex health conditions are not left behind in routine immunization programs.

The likelihood of vaccine deferral or non-recommendation varied substantially across disease categories and vaccine dose numbers. Respiratory and immunological conditions were the most frequently observed diagnoses and were associated with a high proportion of temporary deferrals and non-recommendations. This pattern likely reflects physician concerns about acute exacerbations or immune dysregulation during vaccination and the absence of clear disease-specific national guidance. Early-dose vaccines such as the first and second doses of hepatitis B, polio, and DTaP were almost universally recommended, whereas deferral or non-recommendation was much more common for later doses. This suggests increasing caution among physicians as CSHCN age or accumulate comorbidities, reflecting both uncertainty about the continued necessity of booster doses and concerns about adverse events. Such patterns may be associated with increased vulnerability of high-risk children to vaccine-preventable diseases.

Older age and multimorbidity were independently associated with a higher likelihood of vaccine deferral or non-recommendation, consistent with previous evidence from Zhejiang Province [9]. Our study extends these findings to district-level immunization services, which have not been previously described. Older children with incomplete immunization histories and those with multiple coexisting conditions are often perceived as clinically complex, which may raise concerns about vaccine safety or the applicability of existing guidelines. To promote consistent and timely immunization in these subgroups-particularly in settings with limited access to specialist resources-tailored decision-making protocols and practical clinical support tools are needed.

We also observed striking differences by level of care. Children who received vaccination consultations at primary health centers were more likely to receive a vaccination recommendation than those seen at general hospitals. One possible explanation is that primary health providers in township and subdistrict settings may be more inclined to follow standardized protocols in the absence of explicit contraindications, possibly due to limited access to specialist consultation [17,18]. Alternatively, lower deferral rates in primary health centers may also reflect limited capacity to identify complex contraindications [19]. In contrast, physicians in general hospitals may exercise greater caution, potentially due to heightened clinical responsibility, institutional risk aversion, or policies favoring conservative decision-making. Both patterns carry important clinical and ethical implications—over-deferral may delay essential immunization, whereas under-deferral could expose medically fragile children to avoidable risks. These inter-facility differences illustrate the heterogeneity of real-world clinical decision-making in district-level immunization services. Recognizing such variability is essential for developing harmonized contraindication criteria, standardized provider training, and strengthened decision-support systems that balance caution with equitable access to vaccination and ultimately reduce unwarranted practice variation.

Although more than two-thirds of children whose vaccinations were initially deferred or not recommended eventually received the vaccine, substantial delays were observed and nearly one-third remained unvaccinated at the end of follow-up. Given that only a small fraction (1.5%) of all recommendations were true contraindications, these findings suggest that temporary, precautionary deferral—rather than permanent non-recommendation—was the predominant reason for delayed vaccination [20]. Such patterns highlight the downstream consequences of clinical hesitation and emphasize the need for clearer, disease-specific immunization guidance. Strengthening provider training, implementing decision-support tools, and establishing institutional mechanisms that share responsibility and mitigate perceived legal risks will be critical to improve the consistency and appropriateness of deferral decisions for CSHCN [21].

These findings have important implications for strengthening China’s national immunization framework for children with special health care needs. The observed inconsistencies in vaccination recommendations across disease categories and facility levels underscore the need for clearer, disease-specific guidance and structured provider training at the district level. In practice, such improvements could be achieved by integrating standardized, rule-based decision-support modules into the existing Immunization Information Management System to assist providers in assessing contraindications and scheduling follow-up vaccinations. In regions with limited access to pediatric specialists, teleconsultation functions embedded within provincial or municipal platforms could further enable timely expert input without disrupting service flow. Evidence from this study could inform ongoing efforts to refine China’s expert consensus on CSHCN vaccination and to enhance provider decision support within the national immunization system. Aligning district-level clinical decision-making with evidence-based national guidance may ultimately reduce unwarranted variation and improve equitable vaccine access for medically vulnerable children.

## 5. Limitations

This study has several limitations. First, although all routine vaccination consultation records in the study area were included, selection bias may still exist because the analysis only captured children whose caregivers proactively sought vaccination advice. Children with complex conditions who were managed directly in specialist settings, or whose caregivers did not seek formal consultation, may have had different vaccination outcomes but were not represented in this dataset. Second, clinical diagnoses and vaccination recommendations were based on physician documentation in routine practice, without centralized adjudication or uniform training. This may have introduced interobserver variability and potential misclassification bias arising from differences in disease classification, interpretation of precautionary criteria, and thresholds for vaccine deferral across facilities. In addition, vaccination timeliness in this study was assessed relative to both the official national immunization schedule and physician advice, which may not always align; thus, some delays might reflect conservative clinical judgment rather than true schedule deviation. Accordingly, the reported disease-specific deferral rates should be interpreted as reflecting real-world decision-making patterns rather than adjudicated contraindication rates. Future studies should incorporate standardized case definitions, unified provider training, and independent review to improve consistency and comparability across sites. Third, although key covariates were adjusted for in the multivariable model, residual confounding from unmeasured factors such as caregiver vaccine hesitancy, institutional culture, or provider experience remains possible. The study was also not designed to assess the appropriateness or clinical safety of each vaccination recommendation; therefore, differences in deferral rates across facility levels should be interpreted cautiously, as they may reflect both variation in clinical decision thresholds and differences in diagnostic capacity. In addition, although vaccine type (NIP vs. non-NIP) was included as a covariate, other vaccine-specific considerations such as cost, brand availability, and perceived safety profiles were not collected but may influence physicians’ recommendations. Additionally, the study did not examine predictors of catch-up vaccination after deferral because behavioral and contextual factors driving post-deferral uptake were not captured in the dataset. Future studies incorporating richer behavioral data, caregiver cost considerations, and vaccine-specific attributes could help clarify both provider-level and vaccine-level influences on immunization decisions. Finally, as the study was conducted in a single district in China, the findings should be interpreted with caution when applied to regions with different health system capacities or immunization delivery models.

## 6. Conclusions

This study provides the first comprehensively assessment of vaccination recommendation practices for CSHCN in district-level immunization services in China. Nearly half of vaccine doses are being temporarily deferred or not recommended, reflecting substantial inconsistency in clinical decision-making. These findings underscore the urgent need to refine and disseminate disease-specific national vaccination guidelines and to strengthen the standardization of clinical decision-making through regular, structured provider training at the district level. Digital decision-support and teleconsultation tools, while desirable, should serve as complementary aids to improve implementation efficiency and consistency. Strengthening coordination between primary and hospital-based care will be essential to harmonize practices and ensure timely, equitable protection for this medically vulnerable population. As this analysis was based on data from a single district, the findings should be interpreted with caution and validated through multi-site studies across diverse settings to confirm their broader applicability.

## Figures and Tables

**Table 1:** Demographic characteristics of Children with special health care needs in the study (N = 761).

Variables	N (%)
Age (months, median [IQR])	12 [1–47]
0–6	295 (38.8)
7–12	89 (11.7)
13–18	45 (5.9)
19–24	48 (6.3)
25–30	24 (3.2)
31–36	27 (3.5)
>37	233 (30.6)
Sex	
Male	411 (54.0)
Female	350 (46.0)
Disease stage	
Acute phase	258 (33.9)
Stable phase	136 (17.9)
Recovered	178 (23.4)
Other	189 (24.8)
Multimorbidity	
No	741 (97.4)
Yes	20 (2.6)
Healthcare facility type	
General hospital	560 (73.6)
Primary health center	201 (26.4)
Vaccine type	
NIP vaccine	527 (69.3)
non-NIP vaccine	234 (30.7)

**Table 2:** The group distribution of the children’s special health care needs.

Disease Type	Condition	Count (n)	Proportion (%)
Neonatal Disorders (n = 190)	Pathological Neonatal Jaundice	97	51.1
	Premature Infant	62	32.6
	Breast Milk Jaundice	10	5.3
	Others	21	11.0
Congenital Heart Disease (n = 16)	Patent Ductus Arteriosus	5	31.2
	Atrial Septal Defect	5	31.2
	Ventricular Septal Defect	4	25.1
	Others	2	12.5
Immunological Disorders (n = 105)	Eczema	52	49.5
	Allergic Rhinitis	20	19.0
	History of Adverse Vaccine Reaction	7	6.7
	Others	26	24.8
Respiratory Diseases (n = 317)	Acute Upper Respiratory Infection	207	65.3
	Common Cold	29	9.2
	Neonatal Pneumonia	21	6.6
	Others	60	18.9
Infectious Diseases (n = 62)	Acute Gastroenteritis	17	27.4
	Diarrhea	9	14.5
	Herpetic Pharyngitis	6	9.7
	Others	30	48.4
Chronic Conditions (n = 17)	Nephrotic Syndrome	3	17.6
	Trisomy 21 Syndrome	2	11.8
	Congenital Hypothyroidism	2	11.8
	Others	10	58.8
Neuromuscular Disorders (n = 19)	Epilepsy	8	42.1
	Tic Disorder	3	15.8
	Attention-Deficit Hyperactivity Disorder (ADHD)	2	10.5
	Others	6	31.6
Hematologic and Neoplastic Disorders (n = 23)	Immune Thrombocytopenic Purpura	4	17.4
	Leukemia	4	17.4
	Hemangioma	3	13.0
	Others	12	52.2
Other Special Health Conditions (n = 12)	Trauma	4	33.3
	Rabies Exposure	2	16.7
	Congenital Cataract	1	8.3
	Others	8	66.7

**Table 3:** Comparative summary of vaccine types, dosing status, and recommendations among NIP vaccines.

Vaccine	Dose	Total	Recommended	Temporarily Deferred	Not Recommended	*p*-Value (Fisher Test)
BCG	1	82	78 (95.1%)	4 (4.9%)	0 (0.0%)	-
HBV	1	74	71 (95.9%)	3 (4.1%)	0 (0.0%)	*p* < 0.001
	2	147	141 (95.9%)	6 (4.1%)	0 (0.0%)	
	3	22	10 (45.5%)	11 (50.0%)	1 (4.5%)	
	4	5	0 (0%)	4 (80%)	1 (20%)	
IPV	1	16	11 (68.8%)	5 (31.2%)	0 (0.0%)	*p* < 0.001
	2	11	7 (63.6%)	4 (36.4%)	0 (0.0%)	
	3	3	3 (100.0%)	0 (0.0%)	0 (0.0%)	
	4	34	3 (8.8%)	31 (91.2%)	0 (0.0%)	
OPV	1	7	7 (100.0%)	0 (0.0%)	0 (0.0%)	*p* < 0.001
	2	22	4 (18.2%)	16 (72.7%)	2 (9.1%)	
DTaP	1	11	6 (54.5%)	5 (45.5%)	0 (0%)	*p* = 0.004
	2	10	6 (60%)	3 (30%)	1 (10%)	
	3	12	10 (83.3%)	2 (16.7%)	0 (0.0%)	
	4	19	3 (15.8%)	14 (73.7%)	2 (10.5%)	
DT	1	21	4 (19.0%)	16 (76.2%)	1 (4.8%)	-
MMR	1	24	14 (58.3%)	10 (41.7%)	0 (0.0%)	*p* = 0.157
	2	35	12 (34.3%)	21 (60.0%)	2 (5.7%)	
JE_L	1	15	6 (40%)	8 (53.3%)	1 (6.7%)	*p* = 0.634
	2	21	5 (23.8%)	15 (71.4%)	1 (4.8%)	
JE_I	1	3	2 (66.7%)	1 (33.3%)	0 (0.0%)	*p* = 1.00
	2	1	1 (100.0%)	0 (0.0%)	0 (0.0%)	
MenA	1	16	5 (31.2%)	11 (68.8%)	0 (0.0%)	*p* = 1.00
	2	22	7 (31.8%)	15 (68.2%)	0 (0.0%)	
MenAC	1	43	11 (25.6%)	31 (72.1%)	1 (2.3%)	*p* = 0.029
	2	42	3 (7.1%)	39 (92.9%)	0 (0.0%)	
HepA_L	1	22	5 (22.7%)	16 (72.7%)	1 (4.5%)	-
HepA_I	1	9	7 (77.8%)	2 (22.2%)	0 (0.0%)	*p* = 0.197
	2	11	5 (45.5%)	6 (54.5%)	0 (0.0%)	

**Table 4:** Vaccination coverage of NIP vaccines with temporarily deferred or not recommended.

Vaccines Type	N (%)
BCG (n = 4)	
actual vaccination	4 (100.0)
not vaccinated	0 (0.0)
HBV (n = 26)	
actual vaccination	20 (76.9)
not vaccinated	6 (23.1)
IPV (n= 40)	
actual vaccination	30 (75.0)
not vaccinated	10 (25.0)
OPV (n = 18)	
actual vaccination	9 (50.0)
not vaccinated	9 (50.0)
DTaP (n = 27)	
actual vaccination	21 (77.8)
not vaccinated	6 (22.2)
DT (n = 17)	
actual vaccination	12 (70.6)
not vaccinated	5 (29.4)
MMR (n = 33)	
actual vaccination	24 (72.7)
not vaccinated	9 (27.3)
JE_L (n = 25)	
actual vaccination	15 (60.0)
not vaccinated	10 (40.0)
JE_I (n = 1)	
actual vaccination	1 (100.0)
not vaccinated	0 (0.0)
MenA (n = 26)	
actual vaccination	22 (84.6)
not vaccinated	4 (15.4)
MenAC (n = 71)	
actual vaccination	38 (53.5)
not vaccinated	33 (46.5)
HepA_L (n = 17)	
actual vaccination	13 (76.5)
not vaccinated	4 (23.6)
HepA_I (n = 8)	
actual vaccination	1 (12.5)
not vaccinated	7 (87.5)

**Table 5:** Factors associated with vaccination recommendations among Children with special health care needs in the study.

Variables	Vaccination Recommendations with Temporarily Deferred or Not Recommended			
	OR	95% CI	aOR	95% CI
Age (months, ref: 0–6)				
7–12	9.28 ***	(5.27–16.66)	5.26 ***	(2.30–12.33)
>13	29.88 ***	(19.18–48.19)	13.48 ***	(6.69–28.34)
Sex (Female vs. Male)	0.97	(0.73–1.29)	0.82	(0.51–1.33)
Disease stage (Acute vs. Stable/Recovered/Other)	0.06 **	(0.01–0.28)	0.23	(0.01–1.54)
Multimorbidity (Yes vs. No)	40.15 ***	(24.88–68.13)	20.68 ***	(11.26–40.10)
Disease type (ref: Neonatal Disorders)				
Congenital Heart Disease	3.25	(0.46–14.58)	1.20	(0.14–6.98)
Immunological Disorders	21.49 ***	(10.11–51.5)	1.7	(0.60–5.28)
Respiratory Diseases	67.4 ***	(33.74–154.69)	4.25 **	(1.66–11.69)
Infectious Diseases	19.99 ***	(8.77–50.49)	3.02	(1.01–9.63)
Chronic Conditions	15.92 ***	(4.77–54.03)	2.94	(0.71–12.35)
Neuromuscular Disorders	8.12 ***	(2.21–27.9)	0.78	(0.17–3.41)
Hematologic and Neoplastic Disorders	14.62 ***	(4.9–45.11)	1.43	(0.36–5.75)
Other Special Health Conditions	16.25 ***	(4.08–63.68)	1.03	(0.17–5.99)
Healthcare facility type (Primary health center vs. General hospital)	0.11 ***	(0.07–0.16)	0.26 ***	(0.15–0.45)
Vaccine type (non-NIP vaccine vs. NIP vaccine)	2.09 ***	(1.53–2.86)	0.9	(0.58–1.57)

Note: Statistical significance levels are denoted as follows: *p* < 0.01 (**); *p* < 0.001 (***).

## Data Availability

The data cannot be shared publicly due to concerns about participant confidentiality and privacy. De-identified individual-level data are available for researchers who meet the criteria for access to confidential data and upon approval of a data use proposal.

## Supplementary Materials

The following supporting information can be downloaded at: https://www.mdpi.com/article/10.3390/vaccines13111145/s1, File S1: Special Health Conditions (Operational Definition for CSHCN); Table S1: Comparative summary of vaccine types, dosing status, and recommendations among non-NIP vaccines.
