# Supplementary material for: Vaccination Recommendation Patterns and Associated Factors Among Children with Special Health Care Needs: A Cross-Sectional Study in District-Level Immunization Services in China
Source: Vaccines (Basel). 2025 Nov 7;13(11):1145. doi: 10.3390/vaccines13111145 (PMC12656930; doi:10.3390/vaccines13111145)
Supplement: Supplementary file 1 [file vaccines-13-01145-s001.zip › Supplementary File S1.pdf]

File S1. Special Health Conditions (Operational Definition for CSHCN)

This appendix presents the standardized list of special health conditions used in the EDDC registration form. Items are consolidated according to the nine disease categories shown in Figure 2 of the manuscript. The purpose is to ensure reproducibility and cross-facility consistency in classifying children with special health care needs (CSHCN).

1. Neonatal Disorders

- Preterm infant (gestational age: __ weeks)
- Low birth weight infant (birth weight: __ kg)
- Neonatal intracranial hemorrhage
- Physiologic neonatal jaundice
- Breast milk jaundice
- Pathological neonatal jaundice
- Congenital biliary atresia
- Other neonatal disorders (specify)

For each listed condition, physicians recorded:

(1) presence (Yes/No);

(2) disease stage at consultation: acute exacerbation / stable / recovered / unable to determine / other;

(3) date of initial diagnosis (year, month).

2. Congenital Heart Disease

- Atrial septal defect
- Ventricular septal defect
- Patent ductus arteriosus
- Pulmonary valve stenosis
- Tetralogy of Fallot
- Complete transposition of the great arteries
- Other congenital heart disease (specify)

For each listed condition, physicians recorded:

(1) presence (Yes/No);

(2) disease stage at consultation: acute exacerbation / stable / recovered / other;

(3) date of initial diagnosis (year, month).

3. Immunological Disorders

- Food allergy (specify allergen)

- Pollen allergy
- Drug allergy (specify drug)
- Allergic rhinitis
- Atopic dermatitis (eczema)
- Bronchial asthma
- Allergy to any component of the vaccine to be administered
- History of adverse vaccine reaction
- Henoch–Schönlein purpura (IgA vasculitis)
- Kawasaki disease
- Combined immunodeficiency
- Other well-defined immunodeficiency syndromes
- Predominantly antibody deficiencies
- Immune dysregulation disorders
- Congenital defects of phagocyte number and/or function
- Defects in innate immunity
- Autoinflammatory diseases
- Complement deficiencies
- Primary immunodeficiencies with autoantibodies/phenocopies
- Child born to an HIV-infected mother: HIV-infected child
- Child born to an HIV-infected mother: HIV status unknown
- Child born to an HIV-infected mother: HIV-uninfected child
- Systemic lupus erythematosus
- Juvenile idiopathic arthritis
- Sjögren’s syndrome
- Multiple sclerosis
- Rheumatoid arthritis
- Myasthenia gravis
- Other immunological disease (specify)

For each listed condition, physicians recorded:

(1) presence (Yes/No);

(2) disease stage at consultation: acute exacerbation / stable / recovered / other;

(3) date of initial diagnosis (year, month).

4. Respiratory Diseases

- Acute upper respiratory infection
- Common cold
- Pneumonia
- Other respiratory disease (specify)

For each listed condition, physicians recorded:

(1) presence (Yes/No);

(2) disease stage at consultation: acute exacerbation / stable / recovered / other;

(3) date of initial diagnosis (year, month).

5. Infectious Diseases

- Otitis media
- Sinusitis
- Acute gastroenteritis
- Intra-abdominal infection
- Encephalitis/meningitis
- Perianal abscess
- Congenital infections: syphilis
- Congenital infections: cytomegalovirus
- Congenital infections: rubella virus
- Other infectious disease (specify)

For each listed condition, physicians recorded:

(1) presence (Yes/No);

(2) disease stage at consultation: acute exacerbation / stable / recovered / other;

(3) date of initial diagnosis (year, month).

6. Chronic Conditions

- Congenital hypothyroidism
- Hyperthyroidism
- Trisomy 21 Syndrome
- Phenylketonuria (PKU)
- Methylmalonic acidemia
- Infantile hepatitis syndrome
- Infectious hepatitis
- Autoimmune hepatitis
- Non-alcoholic fatty liver disease
- Hereditary metabolic liver disease
- Nephrotic syndrome
- Urinary tract infection
- Other chronic disease (specify)

For each listed condition, physicians recorded:

(1) presence (Yes/No);

(2) disease stage at consultation: acute exacerbation / stable / recovered / other;

(3) date of initial diagnosis (year, month).

7. Neuromuscular Disorders

- Epilepsy
- Convulsions
- Cerebral palsy
- Other neuromuscular disease (specify)

For each listed condition, physicians recorded:

(1) presence (Yes/No);

(2) disease stage at consultation: acute exacerbation / stable / recovered / other;

(3) date of initial diagnosis (year, month).

8. Hematologic and Neoplastic Disorders

- Anemia (specify type)
- Glucose-6-phosphate dehydrogenase (G6PD) deficiency
- Immune thrombocytopenic purpura (ITP)
- Hemophilia
- Leukemia
- Other hematologic or neoplastic disorder (specify)

For each listed condition, physicians recorded:

(1) presence (Yes/No);

(2) disease stage at consultation: acute exacerbation / stable / recovered / other;

(3) date of initial diagnosis (year, month).

9. Other Special Health Conditions

- Allogeneic hematopoietic stem cell transplantation
- Solid organ transplantation
- Other special health condition (specify)

For each listed condition, physicians recorded:

(1) presence (Yes/No);

(2) disease stage at consultation: acute exacerbation / stable / recovered / other;

(3) date of initial diagnosis (year, month).
