# Supplementary material for: Vaccination Recommendation Patterns and Associated Factors Among Children with Special Health Care Needs: A Cross-Sectional Study in District-Level Immunization Services in China
Source: Vaccines (Basel). 2025 Nov 7;13(11):1145. doi: 10.3390/vaccines13111145 (PMC12656930; doi:10.3390/vaccines13111145)
Supplement: Supplementary file 1 [file vaccines-13-01145-s001.zip › Table S1.pdf]

Table S1. Comparative summary of vaccine types, dosing status, and recommendations among non-NIP vaccines.

Vaccine	Dose	Total	Recommended	Temporarily deferred	Not Recommended	P-value (Fisher test)
MenAC conj	1	4	3 (75.0%)	1 (25.0%)	0 (0.0%)	P=1.00
	2	4	3 (75.0%)	1 (25.0%)	0 (0.0%)	
Influenza	1	52	14 (26.9%)	37 (71.2%)	1 (1.9%)	P=0.699
	2	12	4 (33.3%)	8 (66.7%)	0 (0.0%)	
	3	10	1 (10.0%)	9 (90.0%)	0 (0.0%)	
	4	2	0 (0.0%)	2 (100%)	0 (0.0%)	
Pneumococcal	1	6	4 (66.7%)	2 (33.3%)	0 (0.0%)	P=0.788
	2	5	4 (80.0%)	1 (20.0%)	0 (0.0%)	
	3	1	1 (100.0%)	0 (0.0%)	0 (0.0%)	
	4	4	4 (100.0%)	0 (0.0%)	0 (0.0%)	
Hib	1	2	1 (50.0%)	1 (50.0%)	0 (0.0%)	P=1.00
	2	0	0 (0.0%)	0 (0.0%)	0 (0.0%)	
	3	1	0 (0.0%)	1 (100.0%)	0 (0.0%)	
	4	2	0 (0.0%)	2 (100.0%)	0 (0.0%)	
Rotavirus	1	4	3 (75.0%)	1 (25.0%)	0 (0.0%)	P=1.00
	2	6	5 (83.3%)	1 (16.7%)	0 (0.0%)	
	3	8	6 (75.0%)	2 (25.0%)	0 (0.0%)	
Varicella	1	20	10 (50.0%)	10 (50.0%)	0 (0.0%)	P=0.133
	2	31	8 (25.8%)	23 (74.2%)	0 (0.0%)	
EV71	1	10	5 (50%)	5 (50%)	0 (0.0%)	P=0.432
	2	19	6 (31.6%)	13 (68.4%)	0 (0.0%)	
Pentavalent	1	2	1 (50.0%)	1 (50.0%)	0 (0.0%)	P=1.00
	2	4	2 (50.0%)	2 (50.0%)	0 (0.0%)	
	3	1	1 (100.0%)	0 (0.0%)	0 (0.0%)	
	4	1	0 (0.0%)	1 (100.0%)	0 (0.0%)	
Tetravalent	1	3	3 (100%)	0 (0.0%)	0 (0.0%)	P=0.647
	2	6	6 (100%)	0 (0.0%)	0 (0.0%)	
	3	8	6 (75%)	2 (25%)	0 (0.0%)	
Others	1	27	15 (55.6%)	12 (44.4%)	0 (0.0%)	P=0.346
	2	9	4 (44.4%)	5 (55.6%)	0 (0.0%)	
	3	1	0 (0.0%)	1 (100.0%)	0 (0.0%)	
	4	2	0 (0.0%)	2 (100.0%)	0 (0.0%)	
